# Disclosure of HIV status to sexual partners by people living with HIV

**DOI:** 10.4102/curationis.v38i1.1174

**Published:** 2015-03-23

**Authors:** Gloria T. Tshweneagae, Victoria M. Oss, Tennyson Mgutshini

**Affiliations:** 1 Department of Health Studies, University of South Africa, South Africa

## Abstract

**Background:**

Disclosure of one's HIV status to a sexual partner can have significant health implications. From a health promotion point of view, disclosure is seen as a cornerstone for the prevention of HIV transmission between partners. Despite its importance as a strategy for controlling the spread of HIV, there are challenges that inhibit voluntary disclosure.

**Objectives:**

In exploring factors associated with disclosure of HIV status, the study had two complementary objectives related to: (1) investigation of participants’ views about HIV-positive status disclosure to sexual partners; and (2) a broader identification of factors that influence disclosure of HIV-positive status.

**Method:**

The study explored factors associated with disclosure of the HIV status of people living with HIV to their sexual partners. Purposive sampling was used to select 13 participants living with HIV who attended a wellness clinic. Primary data were collected via an in-depth interview with each of the participants.

**Results:**

The exploration showed that male participants were notably more reluctant to disclose to their sexual partners for fear of rejection; and secrecy was commonly reported around sexual matters. Female participants (who were in the majority) were relatively more willing to disclose their HIV status to their sexual partners. Despite the complexity of disclosure, all participants understood the importance of disclosure to their sexual partners.

**Conclusion:**

There is a need for HIV prevention strategies to focus on men in particular, so as to strengthen disclosure counselling services provided to people living with HIV and to advocate strongly for partner testing.

## Introduction

Disclosure of one's HIV status to a sexual partner can have significant health implications. The first of these is that the negative outcomes of disclosure can be both severe for and detrimental to those affected; and because the second is that low rates of disclosure may lead to increased cases of HIV transmission to others (United States Agency for International Development [USAID] 2006:10–11). In South Africa, new infections are reported every day despite the interventions and efforts put in place to fight the pandemic. Given this trend, strategies to increase disclosure may be a way of reducing new infections.

The Burnet Institute (2010:17) states that HIV infections continue to spread each year. New infections mean that infected people have sex with those who were not previously infected and they then become infected. Ignorance of the sexual partner status has also been shown as the main reason for the spread of HIV.

Whilst disclosure can be an important strategy for controlling the spread of HIV, because of the protective benefits to both individuals and the health system, there are challenges that inhibit voluntary disclosure (Maman *et al*. 2001); these challenges require measures to help people living with HIV (PLWH) to deal with them (Adedimeji 2010:17). Voluntary HIV counselling and Testing (VHCT) has also proved to be helpful in assisting PLWH to disclose their HIV status to their significant other persons (Gatta & Thupayagale-Tshweneagae 2012).

VHCT provides access to structured therapeutic intervention so that affected individuals and couples can make informed health-promoting choices about being tested for HIV (Shangula 2006:23). Along with testing, HIV counselling plays an important role as a prevention intervention. Initial awareness about one's status serves as an important first step that allows affected individuals a chance to better understand the health implications of their HIV status and make informed choices for the future. To this end, the development of affordable and effective medical care for people living with HIV is urgently needed to improve access and quality of service because of increased demand (IPPF South Asia Regional Office and UNFPA 2004:6).

### Problem statement

Contemporary literary sources show that there is limited disclosure of one's HIV status to a sexual partner (Almeleh 2004:139; Gari, Habte & Markos 2010:88). Partners who are HIV-positive usually disclose their status to other family members, such as parents and siblings, but rarely to their sexual partners (Harris & Touray 2004:12; Horn 2010:1). The principal investigator, at her place of work in a wellness clinic, anecdotally found that patients, especially women who tested HIV-positive, were reluctant to share their diagnosis with their sexual partners, preferring rather to tell their parents or siblings.

In 2008, the Northern Cape had a lower prevalence rate of HIV compared with other provinces in South Africa (Department of Health 2008). However, a study by Isaacs and Hara (2008) on mainstreaming of HIV into South African Fisheries Policy, showed that the population studied in the Northern Cape were not aware of the underlying contributory factors for HIV (Isaacs & Hara 2008:8). One of these factors may be the reluctance to disclose one's HIV status to sexual partners.

#### Purpose of the study

The purpose of the study was to explore factors associated with disclosure of HIV status by PLWH to their sexual partners with the aim of improving HIV interventions for PLWH.

#### Research objectives

There were two objectives for this study, namely:

- To investigate participants’ views about HIV-positive status disclosure to sexual partners at the Galeshewe Day Hospital Wellness Clinic in Kimberly (in the Northern Cape Province).
- To identify factors that influence disclosure of HIV-positive status to sexual partners.

#### Research questions

The study purported to answer two research questions as:

- What are the participants’ views about HIV-positive status disclosure to sexual partners?
- What are the factors that influence disclosure of HIV-positive status to sexual partners?

### Contribution to field

Factors associated with disclosure of HIV status to sexual partners would assist in improving planning for HIV interventions amongst PLWH. Disclosure of HIV status offers considerable benefits from both an individual and a public health perspective (World Health Organization [WHO] 2003). If measures are put in place to increase disclosure amongst PLWH to sexual partners, this might contribute to a reduction in the rate of new infections, because when boyfriends, girlfriends, husbands and wives know about the status of their partners, they will take measures to protect themselves at all times. This could then contribute to a lower mortality rate and could also prolong life expectations and productive active participation in daily life, knowing that significant other people know about one's HIV status.

The burden of guilt and secrecy associated with non-disclosure will be minimised. Disclosure also encourages healthy attitudes as partners come to understand and approach safer practices, such as abstinence, sticking to one sexual partner and using protection, amongst others. The life of both the infected and affected can thus be prolonged, as all of the above factors work synergistically to not only prolong their lives but also to promote both their relevance and productive participation in daily activities, at home amongst their families and in society.

## Research methods and design

### Design

A qualitative study using in-depth interviews was conducted with 13 purposively-selected participants living with HIV. The target population was made of both male and female participants living with HIV, who attended the wellness clinic at Galeshewe Day Hospital in the Northern Cape and who were between the ages of 18 and 45 years. This age group was selected because, according to Avert (2010:47), almost one in three women aged between 25 and 29 years is affected and over a quarter of men aged between 30 and 34 years is affected in sub-Saharan Africa. Generally the population most affected globally is between the ages of 15 and 49 years (UNAIDS 2010:29). The lower limit of the age group for this study was chosen based on the age of consent in South Africa.

### Data collection method

Data were collected over a three-week index period. Community counsellors at the wellness clinic assisted with recruitment of participants following the eligibility criteria, after which the primary researcher contacted participants telephonically to secure appointments. Out of 18 recruited participants, one declined, one did not answer her phone and the other one had left the village by the time of contact. Two more did not show up for the assigned time of the interview. The remaining 13 participants were seen on separate dates, depending on their availability. The principal investigator explained the consent form to each participant before the actual interview. A list of questions was prepared to guide the interview but questions were made open to allow participants the freedom to expand on them.

The interviews were conducted in Tswana because the participants were more familiar and comfortable with the language. Although the consent form was in English, the researcher explained it clearly in Tswana. Each participant was interviewed at the identified private room at the hospital. The interviews lasted between 30 and 45 minutes, depending on the participant.

Interviews were tape recorded with the permission of the participants and field notes were also made during the interview.

### Data analysis

Data from each component of the study were analysed using Joubert and Ehrlich’ s (2007) principles of content analysis. According to Joubert and Ehrlich (2007:324), the first step of data analysis entailed the search for broader categories from the transcripts from the audiotaped interviews so as to acquire a sense of the way in which participants expressed the factors associated with disclosure. Tabulation and frequencies were used to order similar categories and, in the final step, thematic strands were woven together into an integrated picture of the factors associated with disclosure of HIV status to sexual partners.

## Results and discussion

Two major themes emerged from the study, namely, support and sexuality. The support theme had two categories – partner reaction to disclosure and partner support – and the sexuality theme had one category, namely, the desire to have children.

The findings are presented as demographic data and as themes that emerged from the study.

### Demographic data of study participants

In the present study, the most represented age group (*n* = 5) was the 36- to 40-year olds, followed by the 26–30-year age groups, represented by three participants. There were two participants in the 31–35 and 41–45 age groups and one in the 20–25 age group (Table 1).

**TABLE 1:** Demographic data of participants.

Demographic variables	Number	Percentage
**Age**		
20–25	1	7
26–30	3	24
31–35	2	15
36–40	5	39
41–45	2	15
**Gender**		
Female	10	77
Male	3	23
**Marital status**		
Single	9	69
Customary marriage	1	8
Civil marriage	1	8
Cohabiting	2	15
**Educational level**		
Never attended school	0	0
Up to Grade 7	1	8
Grade 8 to 10	1	8
Grade 11 to 12	11	84
**Employment status**		
Unemployed	12	92
Employed	1	8
**Number of children**		
0	3	23
1–2	8	62
3	2	15
6 or more	0	0

The sample confirms Avert’ s (2010: 3) assertion that almost one in three women aged 25–29 and more than a quarter of men aged 30–34 are affected in sub-Saharan Africa. The Third South African National HIV Communication Survey (John Hopkins Health and Education in South Africa 2012:1) also asserts that HIV prevalence peaks at 32.7% amongst women aged 25–29 years, whilst for men, it peaks at 25.8% in the 30–34-year age group. This is also supported by the literature, which indicates that generally, the population most affected globally are the 15–49 age group (UNAIDS 2010:5).

The majority of the sampled participants (*n* = 9) were single. Two were married according to either civil law or customary (marriage) law and two were cohabiting. This is in contrast with the study performed by Budlender, Chobokoane and Simelane (2004:5), which found that cohabitation was a real problem for most poor women. This did not seem to be the case in the present study as most of the female participants (*n* = 10) were unemployed but did not opt to cohabit.

The majority of participants in the study were unemployed (*n* = 12). Two of the participants received social grants and most were dependent on their parents.

The majority of the participants (*n* = 10) were women. Eleven of the participants were literate and had attended school up to Grade 12, whereas two of the participants had finished school with either Grade 7 or Grade 8.

The majority of participants (*n* = 8) had one or 2 children, only 2 participants had 3 children and 3 out of 13 participants did not have children because of a fear of being in a relationship. According to the WHO (2010:2), the fertility rate is at 2.5 for children born per woman. South Africa has experienced a 40% decline in fertility from pre-transition. It has also emerged in the survey done by the South African Institute of Race Relations (2009:51) that South African women are having fewer and fewer children.

### Themes and categories generated from the study

Two themes, one of which had two categories, emerged from the qualitative data. Each of the themes will be discussed and the participants’ narratives will be presented in order to support the findings. The themes and categories generated from the data are displayed in Table 2.

**TABLE 2:** Themes and categories generated from the study.

Themes	Categories
Support	Partner reaction to disclosure. Partner support.
Sexuality	Desire to have children.

#### Theme 1: Support

The first theme that emerged was ‘support’, which emerged as two discernable categories, namely, ‘partner reaction to disclosure’ and ‘partner support’.

**Category 1: Partner reaction to disclosure:** The majority of the participants (*n* = 9) had disclosed their status to their sexual partners. Three participants had not disclosed because they were not involved in relationships at the time of the study. However, these participants maintained that if they were in relationships, they would tell their partners. One participant did not disclose his status because he feared rejection. The variety of partner reactions, covering fear, ignorance, anger, secrecy, rejection, silence and acceptance, is displayed in Table 3.

**TABLE 3:** Partner reaction to disclosure.

Partner reaction	Participants’ narratives
Fear	‘I feel that informing a new partner about my status will scare them away. One partner freaked after I informed her about my status and she stopped contacting me, I later told her I was joking and then she came back.’ (P1, Male, 28 years old)
Ignorance	‘When I told him, he just took it lightly. He would sometimes bribe me into not using a condom. The day I informed him, we did not have condoms but he insisted on having unprotected sex.’ (P2, Female, 42 years old)
Anger	‘He was furious with me at first after I informed him about my status. He deserted me for one month but later accepted and started supporting me and reminding me to take treatment.’ (P6, Female, 35 years old)
Secrecy	‘When I told him, he said he is HIV-positive. He did not inform me before that he was HIV-positive.’ (P3, Female, 32 years old) ‘We did couple testing. He remained quiet for some time after receiving the result and later he began to be supportive and confessed that he had a relationship with a partner who had died because of AIDS.’ (P4, Female, 41 years old)
Rejection	‘He did not support the child after I informed him about my status until I applied for maintenance and took a DNA test and we are no more together.’ (P5, Female, 32 years old). ‘My partner left me when the child was one year old after I informed him about my status.’ (P9, Female, 38 years old)
Silence	‘We went for couple testing, and he tested negative. He was quite after testing and later he started to be supportive and confessed that he had a relationship with a partner who died of AIDS.’ (P6, Female, 41 years old)
Acceptance	‘When I disclose to him, I said, “you will be sitting on a mattress next year”, and he said the same thing.’ (P7, Female, 41 years old)

Two of the participants were rejected after disclosing their status. Silence, acceptance and secrecy about the status were also experienced. This study supports Deribe *et al*. (2008:81) and Greeff *et al*.’s (2008:311) findings, which highlighted that although positive effects of disclosure have been identified, such as acceptance and support, there are also potential consequences associated with disclosure, such as abandonment and discrimination.

In general, the study showed some willingness for partners to inform their partners of their status. This finding is well supported in literature. For instance, the Third South African National HIV Communication Survey (John Hopkins Health and Education in South Africa 2012:5) reveals that amongst those who have ever been tested and who know their status, 86% were willing to share their HIV status during the interview. In another study, Iwuagwu (2009:56) reported that all his participants had disclosed their status to their husbands or partners; and Seid *et al*. (2012:100) reported 93.1% disclosure to sexual partners. This finding supports a number of reviewed studies which reported high rates of disclosure amongst females. (Iwuagwu 2009:56; Seid *et al*. 2012:100; UNAIDS 2010:5).

Few participants argued that the fear of being rejected by their partners hindered them from disclosing their status. This is also reported in literature such as the study from USAID/Synergy (2006), which argues that perceived negative reactions discourage people from disclosing their status.

No participant mentioned any form of discrimination, which was indicated by their willingness to disclose their status. This is reinforced by the findings of the Third South African National HIV Communication Survey (John Hopkins Health and Education in South Africa 2012:5), which alluded to the fact that social stigma is gradually disappearing, largely because of HIV communication programmes and a cumulative behaviour change in South Africa over the last 10 years. Factors that the participants felt would disable their disclosure included not knowing where to start, difficult partners and fear of rejection.

Although few participants feared disclosure, they were in agreement in their acknowledgement that it was a difficult process. They communicated the need to disclose in order to protect their partners from contracting the disease. This was seen as a way of ensuring more mutually supportive relationships between partners.

The other interesting finding of the study was the reluctance of men to disclose their status to their sexual partners. Men remained silent about their status until their partner(s) tested positive – and that is the only time when they admitted that they had suspected something regarding their own status all along. This was supported by Seid *et al*. (2012:102) who also state that silence in male partners could be indicative of the fact that they were already infected. This assertion is consistent with the findings at Jima University Hospital in Ethiopia, where it emerged from the study that couples testing helps to facilitate disclosure (Erku, Megabiaw & Wubshet 2012:86).

An interesting finding from the study was that participants used cultural explanations to disclose their HIV status to their partners. Culture pervades all areas of life, including the explanation for HIV and its disclosure to partners. The statements from participants that could only be understood by persons of the same culture are:

- Laying on the mattress.
- Let's allow it to happen.
- Lighting the candle.

The three statements can only be understood to mean ‘death’ by the Tswana ethnic groups and are an indirect way of saying, ‘I am HIV positive’. Literature calls it cultural diversity (Turan *et al*. 2014:3) and it is a clear demonstration that in every culture there are basic standards of social interaction.

**Category 2: Partner support:** Six of the participants received support from partners after disclosure. Although the partners had an initial negative reaction to the news, they later accepted the information and offered their partners support. Quotes that supported this category are as follows:

‘He was furious with me at first. He deserted me for one month but later accepted and started to support me and even reminded me to take treatment.’ (P6, Female, 35 years old)

‘We went for couples testing and he tested negative. He was quiet afterwards, but he later started to be supportive and confessed that he had a relationship with a partner who had died because of AIDS.’ (P3, Female, 41 years old)

The observations within the current study support the findings by Gari *et al*. (2010:86), who concluded that disclosure of HIV status to sexual partners is beneficial in that it may motivate the other partner to seek HIV counselling and testing. It may contribute towards the reduction of risky behaviour and is associated with increased mutual partner support and adherence to antiretroviral therapy (ART).

#### Theme 2: Sexuality

Sexuality is defined by Zimmerman and Dahlberg (2008:71) as a unique (individual) expression of our sexual side which is based on our values, beliefs, experiences and feelings about ourselves in relation to sex.

Three of the participants had difficulties in having sexual relationships as supported by the following quotes:

Fear of relationships: ‘I thought when you are HIV positive you cannot be involved in sexual relationships.’ (P9, Female, 38 years old)

Experience of rape: ‘I tried to pursue relationships when I was in Grade 11 but it was difficult for me to engage in sexual activities because of that experience.’ (P5, Female, 28 years old)

Failure to obtain interest from potential partners: ‘At first, they would give me promises but the next day they would suddenly change their minds. I have given up hope, but next time I will try to pursue people of the same HIV status.’ (P8, Male, 39 years old)

## Ethical considerations

Adherence to sound ethical principles including the unequivocal protection of respondents was maintained throughout this study. In advance of study commencement, ethical clearance was applied for and obtained from Research and Ethics Committee of the Department of Health Studies at the University of South Africa (HSDC 60/2011). Site-access approval was obtained from Galeshewe Day Hospital Wellness Clinic after communicating through a formal letter from University of South Africa (UNISA). Participants signed the consent form after a thorough explanation of the study was given. The principle of beneficence was adhered to in this study – the researcher ensured that participants were comfortable and were interviewed in a private room away from noise and prying eyes. Confidentiality, privacy and anonymity were also maintained throughout the study. Participants were reassured that the information they provided would not be traceable to them and that their names would not be mentioned in any document or manuscript emanating from the study. All the transcripts were kept under lock and key in the second author's office. The participants were further informed that they had the right to refuse to participate in the study and that they could withdraw from it at any time during the course of the study.

## Trustworthiness

To facilitate trustworthiness of data, a close adherence to the strategies established by Lincoln and Guba (1985:112), was ensured. Member and peer checking were utilised as the primary interventions to ensure credibility of the data. With regard to the former that is, ‘member checking’, a précis of data collated from the interview was discussed with each participant so as to elicit their views about the accuracy of collected data. With respect to ‘peer checking’, the coder and the researchers coded data independently and later compared their themes for agreement. Disagreements were discussed and clarified until consensus was reached. In order to achieve dependability for the study, the researcher developed an audit trial. The findings were made open to scrutiny by the study supervisor. Nominated samples and dense descriptions were provided to ensure transferability. The researchers visited the study site three times before data collection so as to acquaint themselves with the prospective participants and develop a trusting relationship. Visits to the study site were also done frequently by the first author in order to maintain contact with the participants. The first author works in the area, thus prolonged engagement with the field and the participants occurred automatically.

## Limitations of the study

The limitations of this study include sampling and dissemination issues. The sample was very small and dominated by more women than men, which makes generalisation of the findings difficult. Although not generalisable, our findings reflect those of others in settings such as Botswana and Zimbabwe. Data collection was completed approximately two years ago and dissemination of this finding will only be done this year, which could be a potential problem as a lot of things might have changed in the intervening years.

## Recommendations

HIV counsellors should be encouraged to discuss the importance of disclosure to their patients. From the findings of this study, it is evident that disclosure comes as a shock to those on the receiving end. Men should also be encouraged to take an active part in HIV issues so that their voices could be heard. There is a need for more research on disclosure of HIV status to sexual partners. The envisaged study should include more men and have more participants.

## Conclusion

The purpose of the study was to explore factors associated with disclosure of HIV status by PLWH to their sexual partners. In-depth interviews were conducted with 13 PLWH men and women between the ages of 18 and 45. This study has shown an increase in percentage of disclosure, as well as a willingness to disclose on the part of those who have not yet disclosed their HIV-positive status to their sexual partners. The benefits of disclosure, as recognised in this study, were support and acceptance.

Gender role is a key aspect in dealing with disclosure of HIV-positive statuses. Women in this study have shown great involvement in disclosure as compared to their male counterparts. Greater involvement on the part of men is still needed.
